# Retinal artery occlusion as the manifestation of left atrial myxoma: a case report

**DOI:** 10.1186/1471-2415-14-164

**Published:** 2014-12-23

**Authors:** Yingying Yu, Ying Zhu, Aiqiang Dong, Zaoan Su

**Affiliations:** Department of General Internal Medicine, The Second Affiliated Hospital, School of Medicine, Zhejiang University, 88 Jiefang Road, Hangzhou, 310009 China; Department of Cardiac Vascular Surgery, The Second Affiliated Hospital, School of Medicine, Zhejiang University, 88 Jiefang Road, Hangzhou, 310009 China; Department of Ophthalmology, The Second Affiliated Hospital, School of Medicine, Zhejiang University, 88 Jiefang Road, Hangzhou, 310009 China

**Keywords:** Retinal artery occlusion, Atrial myxoma, Visual loss

## Abstract

**Background:**

Retinal artery occlusion caused by myxoma is relatively rare. There are several points that should be taken into consideration to avoid overlooking this disorder.

**Case presentation:**

This case report describes a 43-year-old woman with sudden vision loss in her left eye for 20 days after single sudden syncope. Fundus examination of the left eye showed obscure boundary of optic disc with, reflective dispersion of the retina and poor light reflex of central fovea. A retinal artery occlusion was found in her left eye. Echocardiography revealed a tumor in the left atrium. Visual capacity improved a little during the follow-up.

**Conclusion:**

In any patients with retinal artery occlusion, detailed medical history and echocardiography should be carried out to exclude heart diseases.

## Background

Myxomas are the most common primary cardiac tumours in adults. Most myxomas are found in the left atrium, and typically present with a combination of obstructive, embolic, and constitutional symptoms and thus mimicking many common systemic conditions [1]. We report a case of left atrial myxoma with sudden syncope and retinal artery occlusion as the manifestation. Our case had a single episode of syncope, followed with sudden loss of visual acuity. This history of syncope was overlooked by ophthalmologist in local hospital. Our report will let ophthalmologists be alert to this kind of condition and focus on the detail of medical history.

## Case presentation

**Figure 1:**
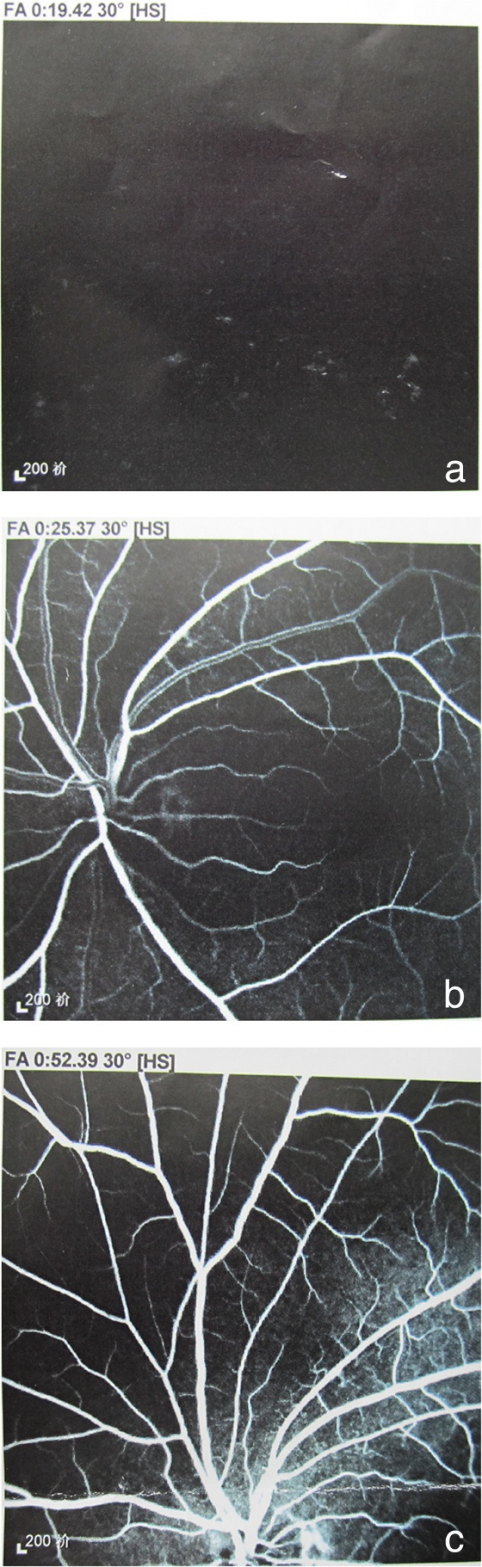
**Fluorescin angiograms imaging: a) 15 seconds showing no choroidal filling; b) 25 seconds showing low fluorescence of the macular and retinal artery was fine; c) 52 seconds showing low filling of the retinal vessels but increased hyperfluorescence of the choroid.**

**Figure 2:**
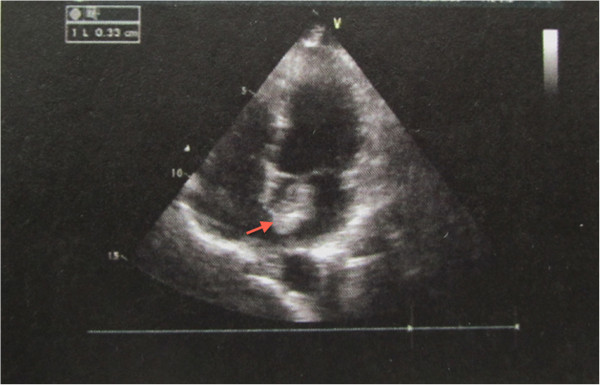
**Echocardiography showing atrial mass during systole (red arrow): septal left atrial side with a hyperechoic mass (1.9 × 2.8 cm), pedunculated in the atrial septum with a pedicle length of 0.3 cm.**

A 43-year-old woman was admitted to the Ophthalmology Department because of vision loss in her left eye for 20 days after single sudden syncope (lasting for about ten seconds; Overlooked by an ophthalmologist at a local hospital). Before admission, she had received treatment by the ophthalmologist at the local hospital, however without improvement. On admission, she was found with visual acuities of 20/25 OD and fingers OS. Intraocular pressure was 16 mmHg OD and 13 mmHg OS. The visual field of the left eye was defected with poor pupillary light reflex. Fundus examination of the left eye showed obscure boundary of optic disc with reflective dispersion of the retina and poor light reflex of central fovea. Fluorescein angiographic imaging showed a left retinal artery occlusion (Figure 1). A transthoracic echocardiography revealed a tumor in the left atrial (Figure 2). She had no prior history of cardiac disease. No ischemic infarct, vertebrobasilar artery insufficiency or other diseases were found after examination. The patient was transferred to the Cardiac Surgical ward for surgery. The physical examination was normal with a blood pressure of 124/70 mmHg and heart rate 57 bpm. Electrocardiogram showed sinus bradycardia, and chest-X-ray showed a normal cardiac silhouette. Bilateral carotid and vertebral artery ultrasound showed no abnormalities. Cerebral MRI revealed no abnormalities. A complete blood count, liver and kidney tests, electrolyte analysis, blood coagulation, myocardial enzymes and blood gas analysis were all normal.

**Figure 3:**
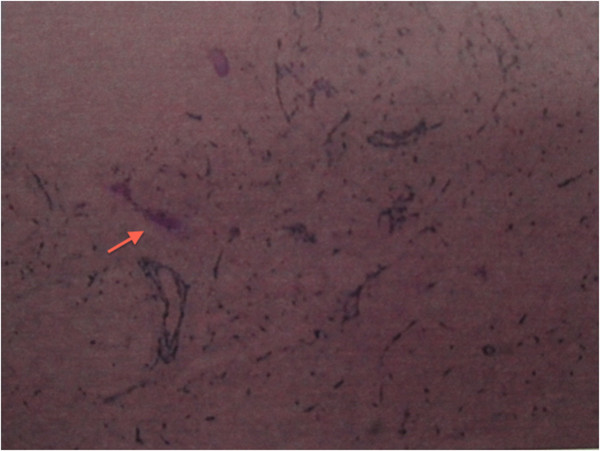
**Tumor histology showing a left cardiac myxoma: mucoid material characteristic of an atrial myxoma.**

The patient underwent minimally invasive percutaneous cardiopulmonary bypass for open heart exploration and the left atrial myxoma. Histology of the excised tissue confirmed left cardiac myxoma (Figure 3). The postoperative course was uneventful and the patient was discharged one week after surgery. She was stable at 12-month follow-up, and her visual acuities improved to 20/400 OS.

## Discussion

Retinal artery occlusion is a terminal artery occlusion caused by acute ischemia of the retina. It may lead to severe irreversible visual impairment. This condition mostly occurs in patients with high blood pressure, heart disease, diabetes or carotid artery atherosclerosis in elderly people [2]. Retinal embolism may consist of cholesterol or fibrin; or they may be calcareous (originating from the heart valves). Or a combination of these different embolic types may occur. Myxomas are rare cardiac tumors and not all myxomas cause retinal emboli. A cardiac myxoma seems to be a rare cause of vascular disturbance in the eye, however, ocular episodes due to emboli of a cardiac tumor have been observed in the literature [3]. Physicians should also be alert that myxoma may cause several other ocular problems, not only central retinal arteries occlusions or branch retinal artery occlusions. Schmidt et al. reviewed that other ocular problems may include homonymous hemianopia or ocular motility disorders, such as diplopia (episode), limitation in upward rotatory nystagmus and horizontal nystagmus. In our case, the woman did not have any other basic diseases; she was stable and no new occlusion happened at 12-month follow-up after operation. Therefore, left atrial myxoma causing retinal artery embolism was considered. In the literature, there are few reported cases of patients with retinal artery occlusions caused by left atrial myxoma since 1990. Schmidt et al. [3] reported a 20-year-old woman diagnosed with branch retinal arterial occlusion (BRAO) in her right eye; echocardiography revealed a myxoma in the left atrium. The author suggested to perform echocardiography to exclude heart diseases in any case of vascular diseases in the eye suspected to be embolic in origin. Salehia et al. reported the case of a 29-year-old patient with an isolated left retinal artery occlusion secondary to a previously undiagnosed left atrial myxoma [3]. And Rafuse et al. [4] reported a 45-year-old woman presented with sudden onset right-side hemiparesis, aphasia and a painful left eye. Examination revealed a bone-white fundus with no perfusion of either the retinal or choroidal circulations. An echocardiogram disclosed a mobile, multilobulated mass attached to the septal wall of the left atrium. Pathological examination of the resected tumour confirmed the diagnosis of endocardial myxoma [4]. Gain et al. also reported a case of middle-aged female patient with burst retinal artery embolism. Echocardiographic examination revealed a left atrial tumor which was confirmed as myxoma following surgery. However, embolisms were also found in other organs.

Notably, our case had a single episode of syncope, followed with sudden loss of visual acuity. This history of syncope was overlooked by ophthalmologist in local hospital. Our report will let ophthalmologists be alert to this kind of condition and focus on the detail of medical history. In conclusion, we report this case of retinal artery occlusion caused by left atrial myxoma. Ophthalmologists should consider the possibility of myxoma in patients with sudden loss of visual acuity; and timely management will be beneficial for better outcomes and prognosis. In addition, our report will let ophthalmologists be alert to this kind of condition and focus on the detail of medical history.

## Conclusion

The present case of retinal artery occlusion was caused by left atrial myxoma. Further research is required to further elucidate the relationship between retinal artery occlusion and the development of atrial myxoma. We recommend that detailed medical history and routine echocardiography should be considered for patients with retinal artery occlusion.

## Consent

Written informed consent was obtained from the patient for publication of this Case report and any accompanying images. A copy of the written consent is available for review by the Editor of this journal.
